# Why a new online open access journal in the field of clinical and epidemiological research in mental health?

**DOI:** 10.1186/1745-0179-1-1

**Published:** 2005-04-27

**Authors:** Mauro Giovanni Carta, Maria Carolina Hardoy

**Affiliations:** 1 Division of Psychiatry, Department of Public Health, University of Cagliari, Italy

**Keywords:** psychiatry, epidemiology, clinical practice, mental health, publication, scientific journals, online journals, Open Access, psychiatric journals

## Abstract

*Clinical Practice and Epidemiology in Mental Health *will encompass all aspects of clinical and epidemiological research in psychiatry and mental health, and will aim to build a bridge between clinical and epidemiological research. There are several outstanding mental heath journals covering all aspects of this dynamic field, but none of these journals is devoted to bridging clinical and epidemiological research. The Open Access online distribution of the journal and its inclusion in the leading data bases (such as PubMed Central) will ensure widespread and ready visibility, which are indispensable given the demand for immediate debate and comparison of scientific findings. This launch Editorial provides an overview of the field, and highlights some of the journal policies.

## Introduction

This editorial is designed to introduce the new online journal *Clinical Practice and Epidemiology in Mental Health *(*CPEMH*).

Mental health issues today can rely on medical model based researches, which are capable of providing reliable results following the introduction of descriptive diagnoses in psychiatry. This revolution (implying the use of a common language) took place as recently as the 1970's [1], but is still often deemed inadequate due to the fact that, contrary to other areas of medicine, it is impossible to define psychiatric disorders as etiopathogenetic, anatomopathologic and clinically descriptive entities.

Due to the current situation in psychiatric research, when providing a clinical definition for a disorder made up of a group of cases presenting a similar symptomatology and course, it may prove necessary to establish the degree of homogeneity revealed with regard to environmental and genetic risk factors. It would be of particular interest to evaluate whether the clinical homogeneity could also be extended to "conditions" identified in the general population receiving no psychiatric treatment.

The association between clinical research and epidemiology is of considerable importance in the medical field, and at the current time is possibly even more so for the field of psychiatric research. Clinical Practice and Epidemiology in Mental Health will encompass all aspects of clinical and epidemiological research in psychiatry and mental health, and will aim to build a bridge between clinical and epidemiological research.

*CPEMH *is aimed at clinicians and researchers focused on improving the knowledge base for the diagnosis, prognosis and treatment of mental health conditions; and improving the knowledge concerning frequencies and determinants of mental health conditions in the community and the populations at risk. The journal will also cover health services research and economic aspects of psychiatry, with special attention given to manuscripts presenting new results and methods in the important area of epidemiology of treatments in mental heath, particularly clinical epidemiologic investigation of psychotherapies and pharmaceutical agents.

## Open Access

*CPEMH'*s Open Access policy supports the changes in the way in which articles in mental health can be published [2]. First, all articles are freely and universally accessible online as soon as they are published. Second, the authors hold copyright for their work and grant anyone the right to reproduce and disseminate the article, provided that it is correctly cited and no errors are introduced. Third, a copy of the full text of each Open Access article is permanently archived in an online repository separate from the journal. *CPEMH *articles are archived in the leading databases (such as PubMed Central [3], the US National Library of Medicine's full-text repository of life science literature, and also in repositories at the University of Potsdam [4] in Germany, at INIST [5] in France and in e-Depot [6], the National Library of the Netherlands' digital archive of all electronic publications).

Open Access has four broad benefits for science and the general public. First, all articles become freely and universally accessible online, and so an author's work can be read by anyone at no cost, given that there are no barriers to access their work. This is accentuated by the authors being free to reproduce and distribute their work, for example by placing it on their institution's website. Furthermore, there is evidence that free online articles are more highly cited because of their easier availability [7]. Second, the information available to researchers will not be limited by their library's budget, and the widespread availability of articles will enhance literature searching [8]. Third, publicly funded research will become accessible to all taxpayers (not just those with access to a library with a subscription). As such, Open Access could help to increase public interest in, and support of, research. Note that this public accessibility may become a legal requirement in the USA if the proposed Public Access to Science Act is made law [9]. Similar calls for a move to Open Access of all scientific research have been made recently by the UK government [10]. Fourth, a country's economy will not influence its scientists' ability to access articles because resource-poor countries (and institutions) will be able to read the same material as wealthier ones (although creating access to the Internet is another matter [11]). This is particularly relevant in mental health issues which represent one of the major health priorities throughout the world. Indeed, by the year 2020 depression will be the second leading cause of disability [12]. Mental health research focused on low- and middle-income (LAMI) countries is needed in view of the burden of neuropsychiatric diseases and the deficiency of mental health resources in these countries [13]. Numerous projects set up by international corporations such as the WHO are attempting to develop mental health programmes and researches in developing countries [14].

This is an exciting opportunity to disseminate our science in the new world wide medium of electronic publishing. *Clinical Practice and Epidemiology in Mental Health *looks forward to receiving your submissions.

## General Journal policy and Peer Review policy

*Clinical Practice and Epidemiology in Mental Health *considers the following types of articles:

Research: reports of data from original research.

Reviews: comprehensive, authoritative, descriptions of any subject within the scope of *CPEMH*. These articles are usually written by opinion leaders that have been invited by the Editorial Board.

Short reports: brief reports of data from original research.

Commentaries: short, focused and opinionated articles on any subject within the scope of the journal. These articles are usually related to a contemporary issue, such as recent research findings, and are often written by opinion leaders invited by the Editorial Board.

Case reports: reports of clinical cases that can be educational, describe a diagnostic or therapeutic dilemma, suggest an association, or present an important adverse reaction. All case report articles should be accompanied by written and signed consent to publish the information from the patients or their guardians.

Methodology articles: present a new experimental method, test or procedure. The method described may either be completely new, or may offer a better version of an existing method. The article must describe a demonstrable advance on what is currently available.

Debate articles: present an argument that is not essentially based on practical research. Debate articles can report on all aspects of the subject including sociological and ethical aspects.

Hypotheses: short articles presenting an untested original hypothesis backed solely by previously published results rather than any new evidence. They should outline significant progress in thinking that would also be testable.

Study protocols: describes proposed or ongoing research, providing a detailed account of the hypothesis, rationale, and methodology of the study.

Book reviews: short summaries of the strengths and weaknesses of a book. They should evaluate its overall usefulness to the intended audience.

Manuscripts submitted to the journal will be reviewed by expert reviewers in the field. *CPEMH *will have an open peer review policy. Once the reviewers have provided their feedback, the Editor makes the final recommendation. The Editor will be available to assist authors with content and formatting issues not resolved during the review process. He will also assist the authors of review articles with integration of content with the ATV website (where appropriate). Articles will be published online immediately upon acceptance and soon after listed in PubMed.

Based on the above characteristics and by means of a series of commentaries on the current state of practice of different aspects of mental health, the journal will seek to stimulate discussion of the main psychiatric topics facing clinical and epidemiological research across the scientific community. An editorial board of international renown of about 50 members has been established [15]. The members of the board will contribute to this series of commentaries.

It is no mere chance that the first commentary by Carta and Angst [16] deals with bipolar disorders, arguably a leading issue in current psychiatric research. In spite of the importance of the topic, a number of researchers maintain that it is not adequately taken into account in the medical field, probably due to the marked inconsistency in the available epidemiological data.

## Conclusion

There are several outstanding mental heath journals covering all aspects of this dynamic field, but none of these journals is devoted to bridging clinical and epidemiological research. The Open Access online distribution of the journal and its inclusion in the leading databases (such as PubMed Central) will ensure widespread and ready visibility, which are indispensable given the demand for immediate debate and comparison of scientific findings.

We welcome any advice and input.

## Competing interests

Critics of Open Access often suggest that Editors have a financial incentive to accept articles as more articles means more revenue. However, BioMed Central insists that decisions about a manuscript must be based on the quality of the work, not on whether the article-processing charge can be paid. This policy will certainly apply for *CPEMH *whose authors and readers will benefit from learning about mental health in regions of the world and in particular field of research (e.g psychotherapies vs pharmacoterapies) with limited financial resources. No member of the editorial or advisory boards of *CPEMH *or their Institutions will receive any portion of the article-processing charge.
